# Comprehensive analysis of aberrantly methylated differentially expressed genes and validation of CDC6 in melanoma

**DOI:** 10.1007/s00432-024-05851-x

**Published:** 2024-07-25

**Authors:** Li Liao, Wei Han, Yue Shen, Guoliang Shen

**Affiliations:** 1 Department of Burn and Plastic Surgery, The First Affiliated Hospital of Soochow University, No. 188 Shizi Street, Suzhou, Jiangsu 215000 China; 2 Department of Cosmetic Dermatology, The Affiliated Suzhou Hospital of Nanjing Medical University, Suzhou Municipal Hospital, Gusu School, Nanjing Medical University, 242 Guangji Road, Suzhou, Jiangsu 215008 China; 3 Institute of Regenerative Biology and Medicine, Helmholtz Zentrum München, 81377 Munich, Germany; 4 Faculty of Medicine, Ludwig-Maximilians-Universität, Munich, Germany

**Keywords:** SKCM, Biomarkers, CDC6, Metastatic, DNA methylation

## Abstract

**Background:**

Skin Cutaneous Melanoma (SKCM) is a highly aggressive malignant tumor with a significant increase in mortality upon metastasis. The molecular mechanisms driving melanoma progression remain largely unclear. Recent studies have highlighted the importance of epigenetic alterations, especially DNA methylation, in melanoma development. This study aims to identify and analyze methylation-regulated differentially expressed genes (MeDEGs) in genome-wide profiles between primary and metastatic melanoma.

**Methods:**

Gene expression profiling datasets GSE8401 and gene methylation profiling datasets GSE86355 were collected from the GEO database. Differentially expressed genes (DEGs) and differentially methylated genes (DMGs) were systematically identified. Integration of DEGs and DMGs yielded a set of MeDEGs, which subsequently underwent functional enrichment analysis. The protein-protein interaction (PPI) network was constructed using STRING and visualized using Cytoscape software. Survival analysis was used to select prognostic hub genes. In addition, 37 SKCM and 37 normal skin tissues from the First Affiliated Hospital of Soochow University (FAHSU) were collected for immunohistochemical (IHC) staining and evaluation. Furthermore, DNA methylation patterns of CDC6 were analyzed. To validate these findings, SKCM cell cultures were utilized to elucidate the expression and behavioral characteristics of CDC6. Additionally, gene set enrichment analysis (GSEA) and immune infiltration analysis were conducted for CDC6.

**Results:**

In our study, we discovered 120 hypomethylated-upregulated genes and 212 hypermethylated-downregulated genes. The hypomethylated-upregulated genes were notably associated with biological processes such as spindle assembly checkpoint signaling, mitotic spindle assembly, and negative regulation of mitotic metaphase/anaphase transition. Our pathway analysis revealed significant enrichment in pathways related to dilated cardiomyopathy, amino sugar metabolism, progesterone-mediated oocyte maturation, and chemical carcinogenesis. Conversely, hypermethylated-downregulated genes were found to be enriched in processes like epidermis development, keratinocyte differentiation, and skin development. Additionally, pathway analysis highlighted associations with estrogen signaling, Staphylococcus aureus infection, axon guidance, and arachidonic acid metabolism. Following the establishment of PPI networks and survival analysis, we identified 11 prognostic hub genes: CCNA2, CDC6, CDCA3, CKS2, DTL, HJURP, KRT5, KRT14, KRT15, KRT16, and NEK2. Notably, among the 11 hub genes, our findings indicate that CDC6 plays a pivotal role in enhancing the proliferation, migration, and invasion capabilities of melanoma cells in vitro.

**Conclusions:**

Our comprehensive genomic analyses reveal that genes with aberrant methylation exhibit differential expression during the transition from primary to metastatic melanoma. The identified genes, especially CDC6, which plays a crucial role in enhancing melanoma cell proliferation, migration, and invasion, provide valuable insights into potential methylation-based biomarkers. These findings could contribute significantly to advancing precision medicine in SKCM.

**Supplementary Information:**

The online version contains supplementary material available at 10.1007/s00432-024-05851-x.

## Introduction

Melanoma is a malignant tumor originating from melanocytes, cells primarily responsible for melanin pigment production, predominantly located in the skin but also present in various other organs such as the eyes, ears, leptomeninges, gastrointestinal tract, and oral, genital, and sinonasal mucous membranes(Long et al. 2023). In 2020, the global incidence of melanoma was estimated to be around 325,000(Arnold et al. 2022). Melanoma incidence rates have exhibited a steady increase over the past half-century within fair-skinned populations of European descent. Skin cutaneous melanoma (SKCM) accounts for the vast majority of melanoma diagnoses, representing over 90% of cases, while mucosal and uveal melanomas occur much less frequently, comprising less than 1–5% of cases. Most of the SKCM are associated with ultraviolet radiation (UVR) exposure, while there exist some rarer subtypes unrelated to UVR(Leonardi et al. 2018). The treatment for SKCM has experienced significant evolution over the past decade. Surgery is currently a critical means of treating primary melanoma. For metastatic melanoma, following the introduction of effective systemic therapies, mortality rates in Caucasian populations decreased by 18% within three years(Villani et al. 2022). Notably, checkpoint immunotherapy, a cornerstone of these therapies, has demonstrated efficacy in both adjuvant and neoadjuvant settings, thereby setting a new standard of care. Despite these advancements, the high mortality rates associated with metastatic melanoma continue to pose significant challenges.

DNA methylation, categorized as an epigenetic modification along with histone modifications and non-coding RNAs (ncRNAs), plays a pivotal role in gene expression regulation without altering the DNA sequence(Sarkar et al. 2015). This process involves the covalent addition of a methyl group to cytosine nucleotides, predominantly occurring at CpG dinucleotides, notably within CpG islands (CGIs) at gene promoters. In cancer, including melanoma, aberrant DNA methylation patterns are common, characterized by hypermethylation of CGIs in tumor suppressor gene promoters and global hypomethylation(Aleotti et al. 2021). These alterations contribute to dysregulated gene expression, affecting crucial signaling pathways like MAPK, pRb, PI3K, and p53, implicated in melanoma development and progression. Furthermore, gradual gain in DNA hypermethylation, termed CpG island methylator phenotype (CIMP), correlates with increased tumor aggressiveness(Micevic et al. 2017). Conversely, DNA hypomethylation facilitates tumor progression by inducing genome instability and activating oncogenes, thus playing a significant role in melanoma initiation and evolution. Despite the existence of the single-gene methylation analyses, comprehensive network analyses integrating gene expression, methylation profiles, and associated pathways remain notably scarce.

Over the past years, high-throughput sequencing technologies have unveiled extensive dysregulation in gene regulation associated with various diseases, including melanoma. This approach has been instrumental in identifying numerous oncogenes implicated in the pathological progression of tumors. Bioinformatics technology has emerged as an indispensable tool in tumor research, primarily concentrating on genomics and proteomics. Its aim is to delineate genotypic and phenotypic associations with immune infiltration, tumorigenesis, and tumor progression, thereby guiding the development of targeted therapies. Despite extensive research on methylation changes in SKCM, many aspects remain poorly understood.

In this study, we performed a comparative analysis of primary and metastatic SKCM samples using integrated bioinformatics approaches. We utilized gene expression profiling from high-throughput sequencing and gene methylation profiling via microarray to identify methylation-regulated differentially expressed genes (MeDEGs). Functional enrichment analysis was conducted on these MeDEGs, and protein-protein interaction (PPI) networks along with survival analysis were employed to identify novel prognostic biomarkers and potential therapeutic targets for melanoma. Our study highlights and validates the pro-tumorigenic role of CDC6 in SKCM.

## Materials and methods

### Patients and variables

Melanoma tissues (*n* = 37) and normal skin tissues (*n* = 37) were collected from the First Affiliated Hospital of Soochow University (Suzhou, China) between April 2018 and July 2023. All tissue samples were pathologically confirmed and preserved in 4% paraformaldehyde, and are available from the tissue bank. The study received approval from the Independent Ethics Committee (IEC) of the First Affiliated Hospital of Soochow University, and informed consent was obtained from each subject prior to participation.

### Data mining from public databases

The Gene Expression Omnibus (GEO, https://www.ncbi.nlm.nih.gov/geo/) is a publicly accessible genomics database that houses high-throughput gene expression data for both normal and pathological tissues across various disease types, along with accompanying clinical information(Barrett et al. 2013). In this study, we acquired gene expression profiling datasets from high-throughput sequencing (GSE8401) and microarray-based gene methylation profiling datasets (GSE86355) from GEO. The GSE8401 dataset comprises a total of 31 primary and 52 metastatic melanoma samples, utilizing the GPL96 Affymetrix Human Genome U133A Array platform. Meanwhile, the GSE86355 dataset includes 33 primary and 28 metastatic melanoma samples from SKCM tissues, employing the GPL13534 platform (Illumina HumanMethylation450 BeadChip).

Differentially expressed genes (DEGs) and differentially methylated genes (DMGs) were identified using the GEO2R tool (http://www.ncbi.nlm.nih.gov/geo/geo2r/) with |t| > 2 and adj.*p* < 0.05 as significance criteria. Subsequently, we identified hypomethylation-high expression genes by overlapping upregulated and hypomethylated genes, and hypermethylation-low expression genes by overlapping downregulated and hypermethylated genes. These hypomethylation-high expression genes and hypermethylation-low expression genes were categorized as methylation-regulated differentially expressed genes (MeDEGs) for further analysis.

### Protein-protein interaction (PPI) network construction and module analysis

To investigate functional associations among proteins, we constructed a PPI network of MeDEGs using the STRING database (v 12.0, http://string-db.org) (Franceschini et al. 2013)with interactions having a combined score > 0.4 considered significant. The network was visualized using Cytoscape software (v 3.8.0) (Smoot et al. 2011) and analyzed with the Molecular Complex Detection (MCODE) plug-in (v 1.5.1) (Bandettini et al. 2012). Criteria for identifying significant modules included MCODE scores > 5, degree cut-off of 2, node score cut-off of 0.2, maximum depth of 100, and k-score of 2.

### Functional enrichment of MeDEGs

In this study, we employed Enrichr (https://maayanlab.cloud/Enrichr/), a comprehensive web-based and mobile software application that integrates numerous gene-set libraries, a novel approach for ranking enriched terms, and interactive visualizations of results(Chen et al. 2013). By using Enrichr, we aimed to elucidate the role of MeDEGs in development-related signaling pathways in SKCM and extract pertinent biological attributes, including biological processes (BP), molecular functions (MF), and cellular components (CC), through Gene Ontology (GO) enrichment analysis. P-value < 0.05 was considered statistically significant.

### Survival analysis

Gene Expression Profiling Interactive Analysis (GEPIA, http://gepia.cancer-pku.cn/) is an invaluable online tool offering customizable functionalities by using data from the Genotype-Tissue Expression project (GTEx; https://www.gtexportal.org/home/index.html) and The Cancer Genome Atlas (TCGA; https://tcga-data.nci.nih.gov/tcga/)(Tang et al. 2017). GEPIA facilitates survival analysis based on gene expression levels, employing the log-rank test for hypothesis evaluation.

### Validation and genetic variations of hub genes

We utilized the cBioPortal (http://cbioportal.org), an open-access platform, to explore multidimensional cancer genomics datasets(Cerami et al. 2012). This resource grants access to genetic data from more than 5,000 tumor samples across 20 different cancer studies. Through the cBioPortal, we examined the genetic modifications of central genes and confirmed the survival analysis.

### The human protein atlas

The Human Protein Atlas (https://www.proteinatlas.org/ ) serves as a comprehensive database dedicated to mapping human protein expression profiles across diverse cells, tissues, and organs (Uhlén et al. 2015). In this study, we utilized this resource to extract CDC6 protein expression immunohistochemistry (IHC) images from clinical specimens encompassing both normal skin tissues and samples from patients with SKCM.

### Immunohistochemistry (IHC)

The levels of CDC6 protein expression were determined through IHC staining using a rabbit polyclonal anti-CDC6 antibody (PA5-86121). Two experienced pathologists independently assessed the presence or absence of protein staining in a single FFPE slide, under the supervision of a clinician. Staining intensity was categorized as no staining, weak, moderate, or strong, and was scored on a scale from 0 to 3 (Hofman and Taylor 2013). The extent of staining was evaluated based on the percentage of immunoreactive tumor cells, with scores ranging from 0 to 4 (0%, 1–25%, 26–50%, 51–75%, 76–100%). The total IHC score, obtained by multiplying the intensity and extent scores, ranged from 0 to 12. A score of 0 to 4 indicated negative staining, while a score of 5 to 12 indicated positive staining for each sample.

### Analysis of the DNA methylation data

The Shiny Methylation Analysis Resource Tool (SMART, http://www.bioinfo-zs.com/smartapp/) App is utilized as a user-friendly and intuitive web application designed for comprehensive analysis of DNA methylation data sourced from the TCGA project (Li et al. 2019). In our study, we applied SMART to conduct an in-depth analysis of methylation probes linked to CDC6 in SKCM samples.

### Cell culture and reagents

Human melanoma cell lines A-375 and SK-MEL-28 were obtained from National Collection of Authenticated Cell Cultures (Shanghai, China). The A-375 cell was grown in DMEM medium (Keygene, China, Thermofisher) and SK-MEL-28 cell was grown in 1640 medium (Keygene, China, Thermofisher) containing 10% fetal bovine serum (Every Green) and 1% Penicillin-Streptomycin-Gentamicin Solution (Beyotime). The cells were cultured in a humid atmosphere containing 37 °C with 5% CO2.

### Gene knockdown

siRNAs targeting CDC6 were purchased from Ribobio (Guangzhou, China) (sequences provided in Supplementary Table 1). Cells were seeded in 6-well plates for 24 h before transfection with siRNA. A total of 150 nM of siRNA was transfected in OptiMem medium (Keygene) using Lipofectamine 8000 transfection reagent (Beyotime). The medium was replaced with fresh medium after 24 h. 48 h post-transfection, cells were harvested for further analysis.

### Western blot assay

Cells were harvested by scraping into an SDS sample buffer containing a cocktail of protease inhibitor and PhosSTOP Phosphatase Inhibitor (Beyotime). Western blotting was conducted according to the standard procedure(Sule et al. 2023). The abundance of CDC6 (11640-1-AP, diluted 1:500, Proteintech) and GAPDH (60004-1-Ig, 1:50000, Proteintech) was investigated later.

### Cell counting kit (CCK)-8 assay

CCK-8 kit (Beyotime) was used for cell proliferation assay. Cells were seeded into 96-well plates (1000 cells/well) with 100 µl complete culture medium. After incubation for 1, 2, 3, 4, and 5 days, respectively, 10% CCK-8 solution was added to each well. Then, the cells were cultured for an additional 2 h. The OD values were detected at 450 nm wave length.

### Transwell assay

20,000 cells were seeded on the top of a polycarbonate Transwell filter with 200 µl culture medium in 24-well plates with serum-free medium. The transwell inserts were embedded into complete culture medium. A layer of Matrigel was spread on the filter membrane in the upper surface of the Transwell filter. After culturing for 24 h, the Transwell filter was removed, and cells were fixed with formaldehyde, stained with 1% crystal violet, and photographed.

### Wound scratch assay

500,000 cells were seeded in a six-well plate. A scratch wound was created using a sterile 200-µL pipette tip when cell confluence was up to 90%. Scratch wounds were monitored and photographed at 0 and 24 h.

### Gene set enrichment analysis

Gene Set Enrichment Analysis (GSEA) is a widely employed bioinformatics tool utilized to elucidate the functional roles of target genes in the pathological processes of various diseases(Subramanian et al. 2005). In our study, we employed GSEA (v 4.3.2) to investigate the biological behavior of CDC6 in SKCM, focusing on its functional aspects. Patients with SKCM sourced from The Cancer Genome Atlas (TCGA) were stratified into distinct groups, followed by GSEA analysis utilizing predefined gene sets based on characteristic markers. During GSEA analysis, we utilized 1,000 permutations to assess the significance of enrichment. A p-value less than 0.05, along with a false discovery rate (FDR) below 0.25, were considered indicative of statistically significant differences in gene set enrichment.

### Immune infiltration analysis

In this study, we utilized the Tumor Immune Estimation Resource (TIMER) database, a comprehensive resource facilitating systematic analysis of immune infiltrates across diverse cancer types (Li et al. 2020). We aimed to assess the relationships between CDC6 expression and the infiltration levels of various immune cell types in SKCM using the TIMER platform. Specifically, we evaluated the associations between CDC6 expression and the invasion levels of CD8 + T cells, CD4 + T cells, B cells, neutrophils, macrophages, and dendritic cells in SKCM samples. Moreover, we systematically analyzed the relationship between immune cell infiltration levels and patient survival outcomes.

Furthermore, we identified 28 different types of tumor-infiltrating lymphocytes (TILs) involved in interactions between the immune system and tumors using the integrated repository portal for tumor-immune system interactions (TISIDB, http://cis.hku.hk/TISIDB/index.php)(Ru et al. 2019). Leveraging the CDC6 expression profile, we conducted gene set variation analysis to determine the relative abundance of TILs and assessed the association between CDC6 and TILs using Spearman’s correlation test. Statistically significant associations were determined by p-values less than 0.05, providing insights into the potential involvement of CDC6 in modulating immune responses in SKCM.

## Results

### Clinicopathological characteristics of SKCM patients

A total of 481 SKCM patients were included from the TCGA cohort, with an additional 37 patients from a discovery cohort. The clinicopathological parameters for all patients, including age, gender, Breslow depth, Clark level, T, N, M, and pathological stage, are detailed in Table 1.

**Table 1:** Clinicopathological characteristics of SKCM patients

Characteristics	TCGA cohort (*N* = 481)	Discovery cohort (*N* = 37)
*N (%)*		
*Age*		
≤60 years	258(54.7)	16(43.2)
>60 years	214(45.3)	21(56.8)
Gender		
Male	297(61.9)	20(54.1)
Female	183(38.1)	17(45.9)
*Clark level*		
I	6(1.8)	15(40.5)
II	18(5.5)	17(46.0)
III – IV	246(75.5)	5(13.5)
V	56(17.2)	0(0)
*Breslow depth(mm)*		
≤ 0.75	36(10.2)	7(19.0)
0.76–1.50	65(18.4)	10(27.0)
1.51-4.00	106(30.0)	15(40.5)
>4.00	146(41.4)	5(13.5)
*pT stage*		
T1-T2	121(32.7)	23(62.2)
T3-T4	249(67.3)	14(37.8)
*pN stage*		
N0	236(65.0)	37(100)
N1	75(20.7)	0(0)
N2	52(14.3)	0(0)
*pM stage*		
M0	424(94.4)	37(100)
M1	25(5.6)	0(0)
Pathologic stage		
I- II	233(53.9)	37(100)
III-IV	199(46.1)	0(0)
*Persistent distant metastasis*		
No	217(46.1)	37(100)
Yes	254(53.9)	0(0)

### Identification of methylation-regulated differentially expressed genes in SKCM

**Fig. 1:**
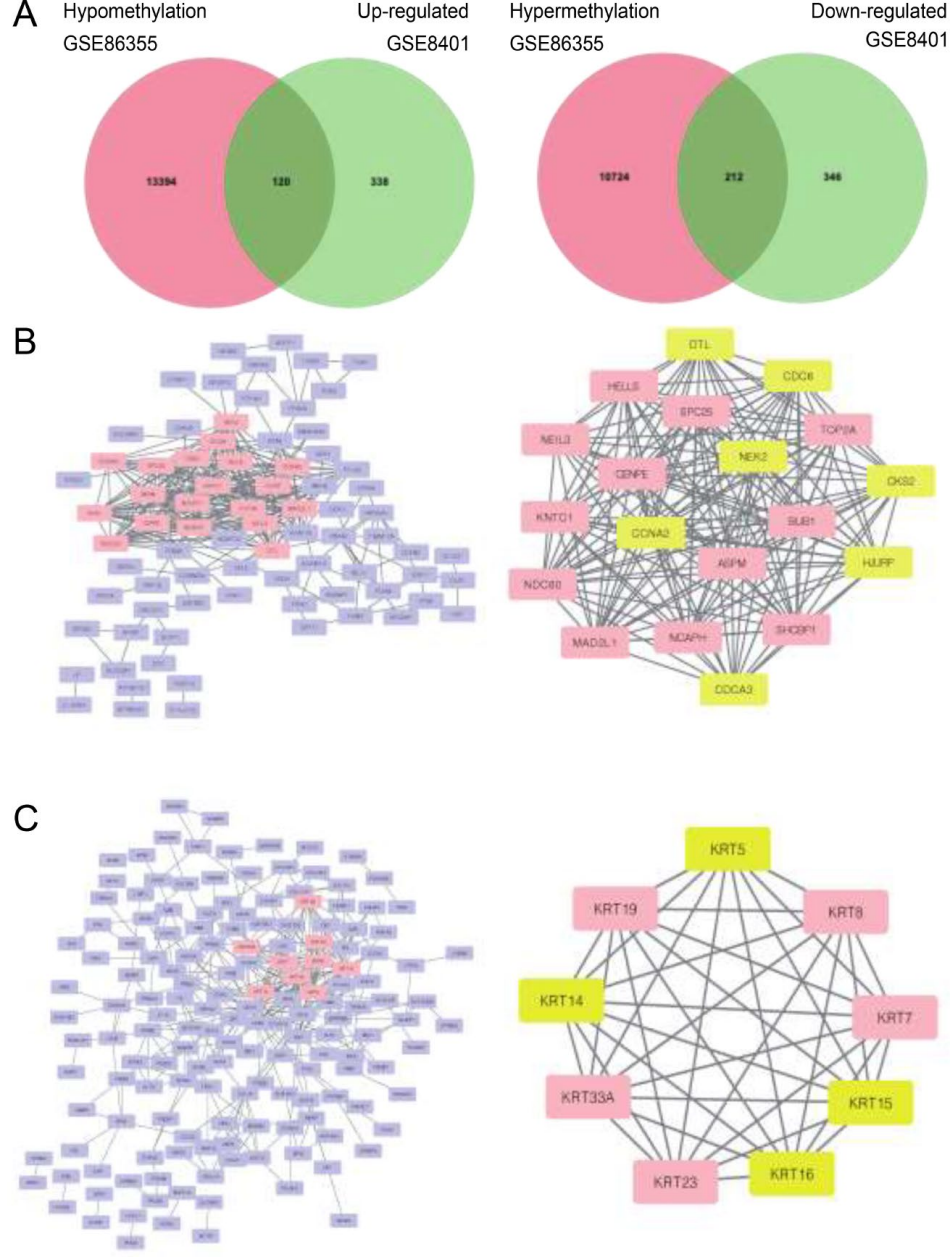
(**A**)Identification of methylation-regulated differentially expressed genes (MeDEGs) in gene expression datasets (GSE86355) and gene methylation datasets (GSE8401). (**B**) PPI network and most related modules of hypomethylated-upregulated genes. (**C**) PPI network and most related modules of hypermethylated-downregulated genes

Using GEO2R, we employed it to identify the DEGs and DMGs in the respective datasets. In GSE8401, 458 genes were up-regulated and 558 genes were down-regulated in gene expression microarray analysis. In GSE86355, 10,936 genes were hypermethylated and 13,514 genes were hypomethylated in gene methylation microarray analysis. Integrating the DEGs and DMGs, we identified 120 hypomethylated, upregulated genes and 212 hypermethylated, downregulated genes, as illustrated in Fig. 1A.

### Protein-protein interaction network establishment and hub genes selection

Utilizing MCODE in Cytoscape, we visualized the PPI network of hypomethylation-upregulated genes and hypermethylation-downregulated genes, depicted in Fig. 1B-C, revealing hub genes within each module. Consequently, DTL, CDC6, NEK2, CKS2, CCNA2, HJURP, CDCA3, TOP2A, HELLS, SPC25, BUB1, SHCBP1, ASPM, NCAPH, MAD2L1, NDC80, KNTC1, NEIL3, and CENPE were identified as hub genes in the hypomethylation-upregulated genes module. Additionally, KRT5, KRT7, KRT8, KRT14, KRT15, KRT16, KRT19, KRT23, and KRT33A were determined as hub genes within the hypermethylation-downregulated genes module.

### Functional enrichment analysis of MeDEGs

**Fig. 2:**
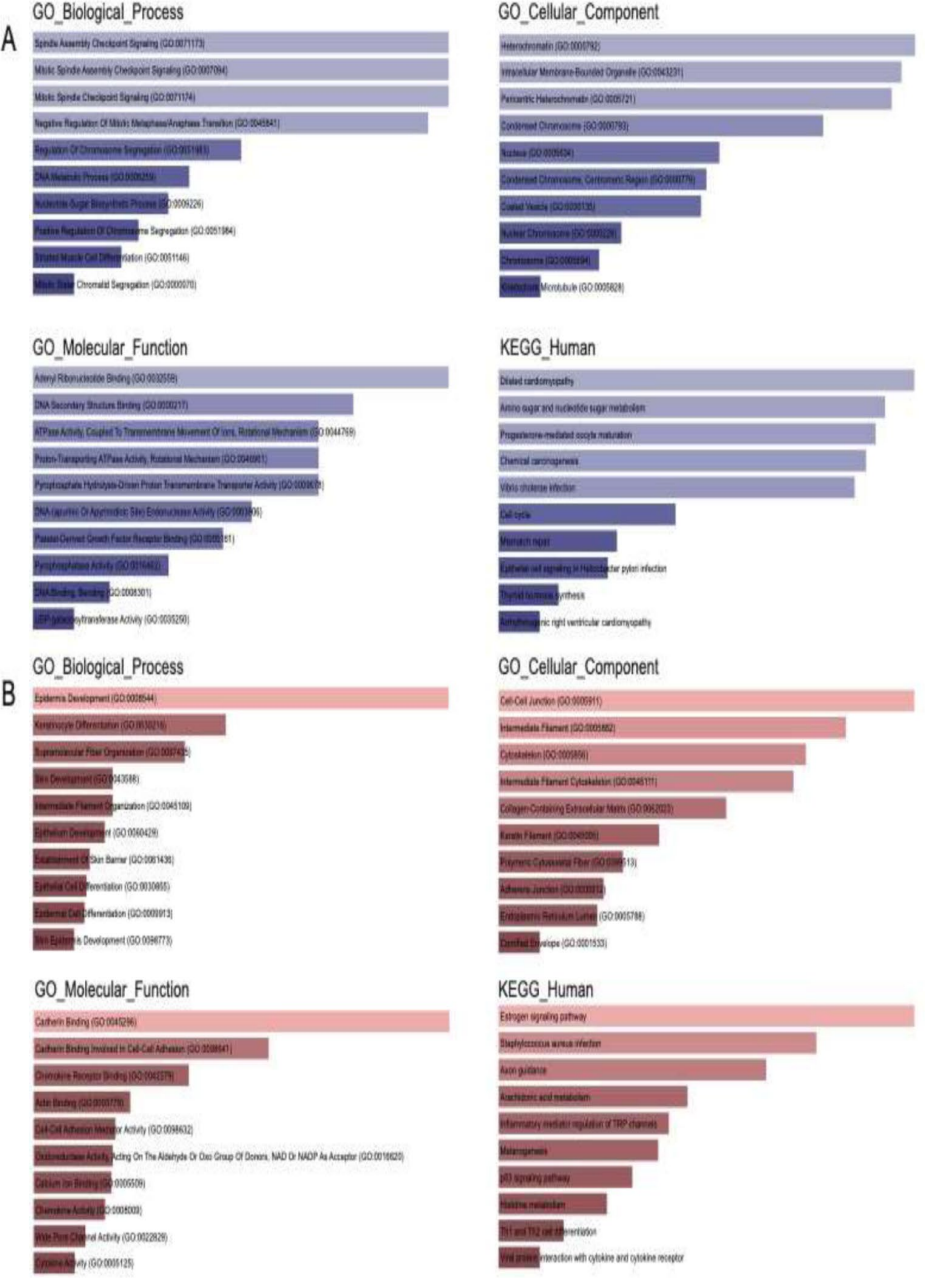
Functional enrichment analysis of MeDEGs (**A**) Hypomethylated-upregulated genes. (**B**) Hypermethylated-downregulated genes

In the Fig. 2A, the results of the GO enrichment analysis and KEGG pathway enrichment for the hypomethylation-upregulated genes were presented, while for hypermethylation-downregulated genes, they were displayed in Fig. 2B. Regarding the hypomethylation-upregulated genes, alterations in biological processes were notably enriched in spindle assembly checkpoint signaling, mitotic spindle assembly checkpoint signaling, mitotic spindle checkpoint signaling, and negative regulation of mitotic metaphase/anaphase transition. Conversely, the hypermethylation-downregulated genes were principally enriched in epidermis development, keratinocyte differentiation, supramolecular fiber organization, and skin development. In terms of cellular components, the hypomethylated, upregulated genes were associated with heterochromatin, intracellular membrane-bounded organelles, pericentric heterochromatin, and condensed chromosomes, while hypermethylated, downregulated genes were linked to cell-cell junctions, intermediate filaments, the cytoskeleton, and intermediate filament cytoskeleton. As for molecular functions, the hypomethylated, upregulated genes exhibited enrichment in adenyl ribonucleotide binding, DNA secondary structure binding, ATPase activity coupled to the transmembrane movement of ions, and proton-transporting ATPase activity. While hypermethylated, downregulated genes were significantly enriched in cadherin binding, cadherin binding involved in cell-cell adhesion, chemokine receptor binding, and actin binding. Regarding KEGG pathway analysis, hypomethylated genes were prominently involved in dilated cardiomyopathy, amino sugar and nucleotide sugar metabolism, progesterone-mediated oocyte maturation, and chemical carcinogenesis. On the other hand, hypermethylated genes were significantly enriched in pathways such as the estrogen signaling pathway, Staphylococcus aureus infection, axon guidance, and arachidonic acid metabolism.

### Survival analysis

**Fig. 3:**
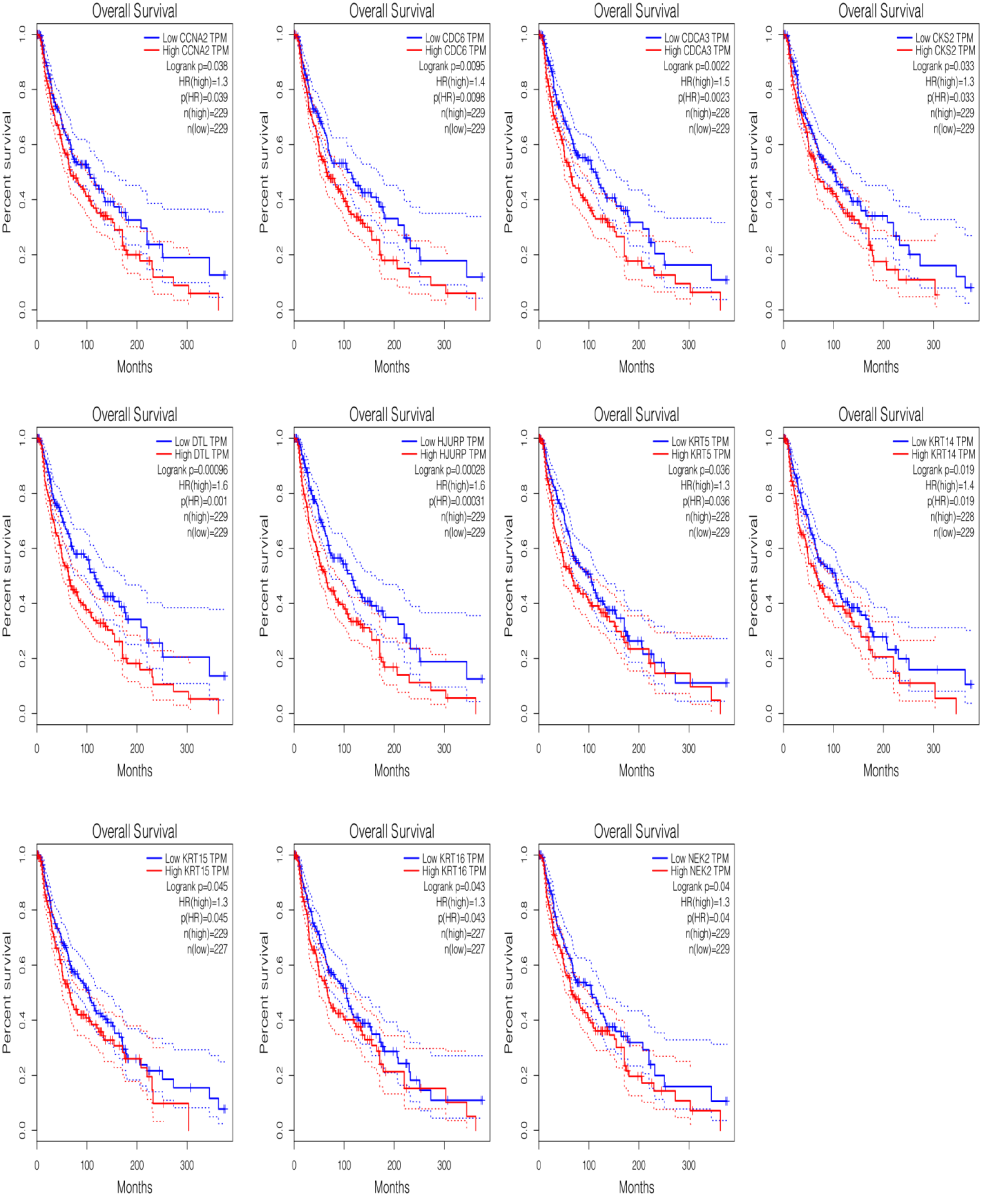
Survival analysis of the hub genes was performed using Kaplan-Meier curve. All of the 11 hub genes showed upregulated expression is markedly associated with significant worse OS in melanoma samples (*P* < 0.05)

The significant survival outcomes of hub genes within the PPI network was illustrated in Fig. 3. By examining the mRNA expression levels of each gene, overall survival data for SKCM patients were obtained. Notably, high mRNA expression of CCNA2 (*p* = 0.039), CDC6 (*p* = 0.0095), CDCA3 (*p* = 0.0023), CKS2 (*p* = 0.033), DTL (*p* = 0.001), HJURP (*p* = 0.00031), KRT5 (*p* = 0.036), KRT14 (*p* = 0.0019), KRT15 (*p* = 0.045), KRT16 (*p* = 0.043), and NEK2 (*p* = 0.04) was significantly associated with poor prognosis for SKCM. Hence, these prognostic hub genes are the focus of further analysis.

### Genetic alterations

**Fig. 4:**
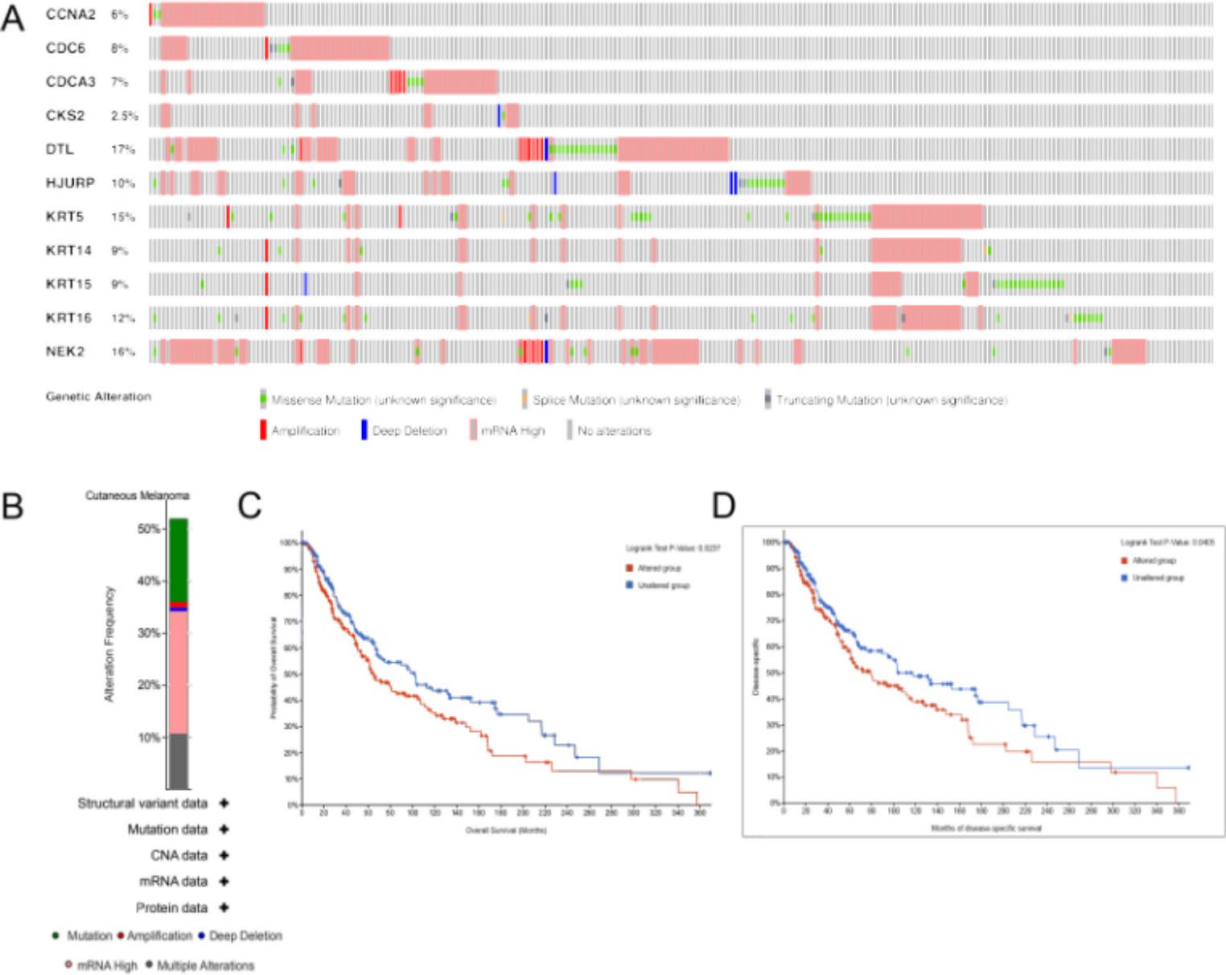
Genetic alteration of 11 hub genes and the validation of survival analysis in the TCGA SKCM study using the cBioPortal database. (**A**) Alteration frequency of hub genes. (**B**) A visual summary of alteration based on a query of 11 hub genes, which was altered in 232 (51.9%) out of 447 sequenced cases/patients. (**C**) The overall survival analysis of 11 hub genes. (**D**) The disease-specific survival analysis of 11 hub genes

Furthermore, we delved into the genetic alterations of the 11 prognostic hub genes by utilizing the cBioPortal tool. It was revealed that DTL (17%) and NEK2 (16%) exhibited the highest frequency of alterations among all prognostic hub genes, encompassing mRNA high, amplification, deep deletion, fusion, and missense mutations (Fig. 4A). These alterations were observed in 232 (51.9%) out of 447 sequenced cases/patients (Fig. 4B). Moreover, both overall survival and disease-specific survival analyses exhibited statistical significance (*p* = 0.0237, *p* = 0.0405), indicating that the altered group experienced a worse prognosis (Fig. 4C-D).

### Transcriptome, protein expressions and DNA methylation status of CDC6

**Fig. 5:**
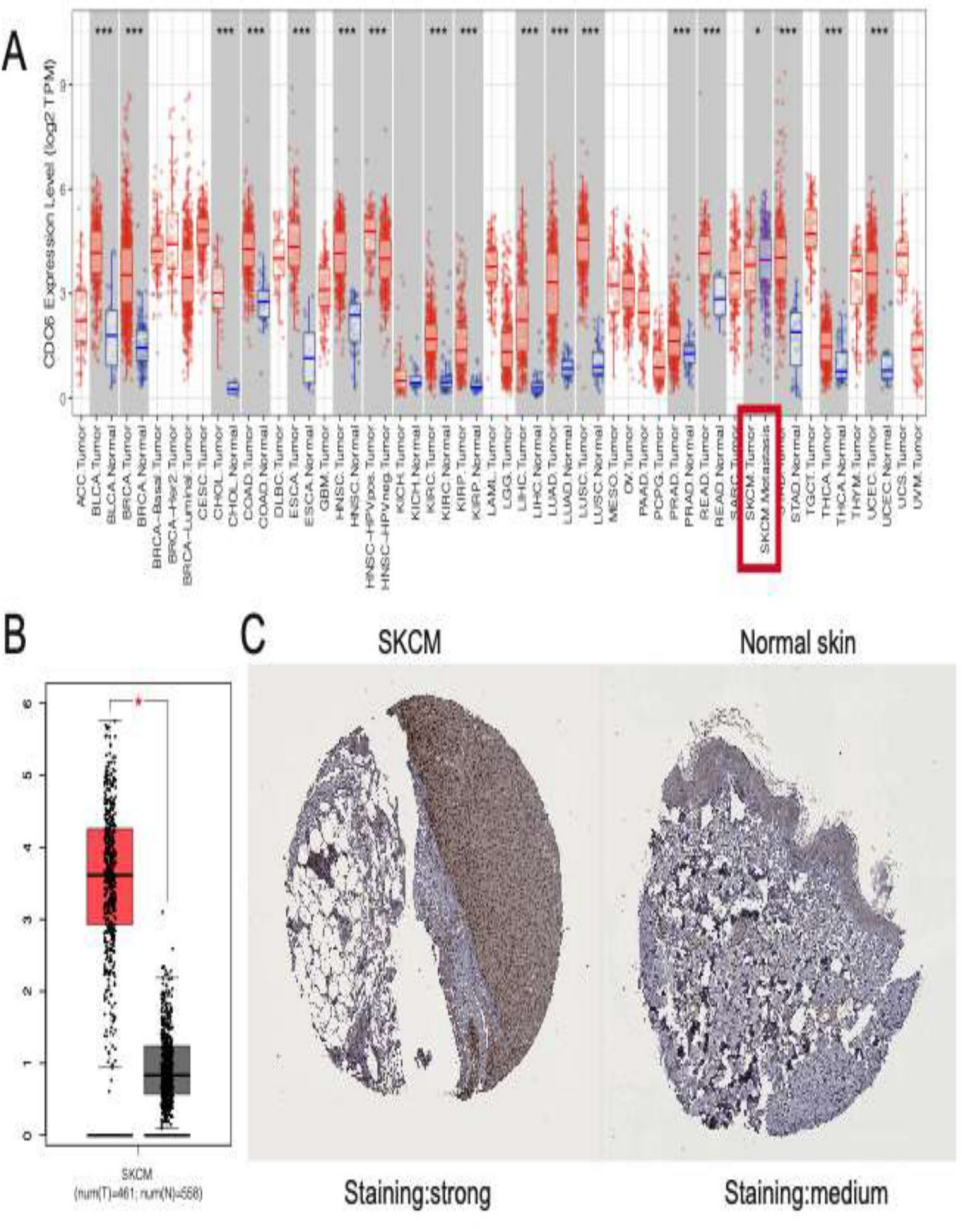
Transcriptome and protein expressions of CDC6 in SKCM showed that CDC6 is upregulated at both the transcriptomic and protein levels

After reviewing the literature, we focused on conducting further research on CDC6. As depicted in Fig. 5A, the transcriptomic level of CDC6 increased in metastatic SKCM compared to primary samples. Furthermore, when compared to normal skin samples, CDC6 also exhibited upregulation (Fig. 5B). Additionally, immunohistochemistry (IHC) images demonstrated that the protein expression followed the same trend as the gene expression level, indicating that CDC6 is upregulated at both the transcriptomic and protein levels. To further validate the results from public database, IHC staining from the discovery cohort was performed. IHC analysis revealed that CDC6 protein expression was significantly higher in SKCM tissues compared to normal skin tissues. These findings, along with the IHC score plots (*p* < 0.0001), are presented in Supplementary Fig. 1.

**Fig. 6:**
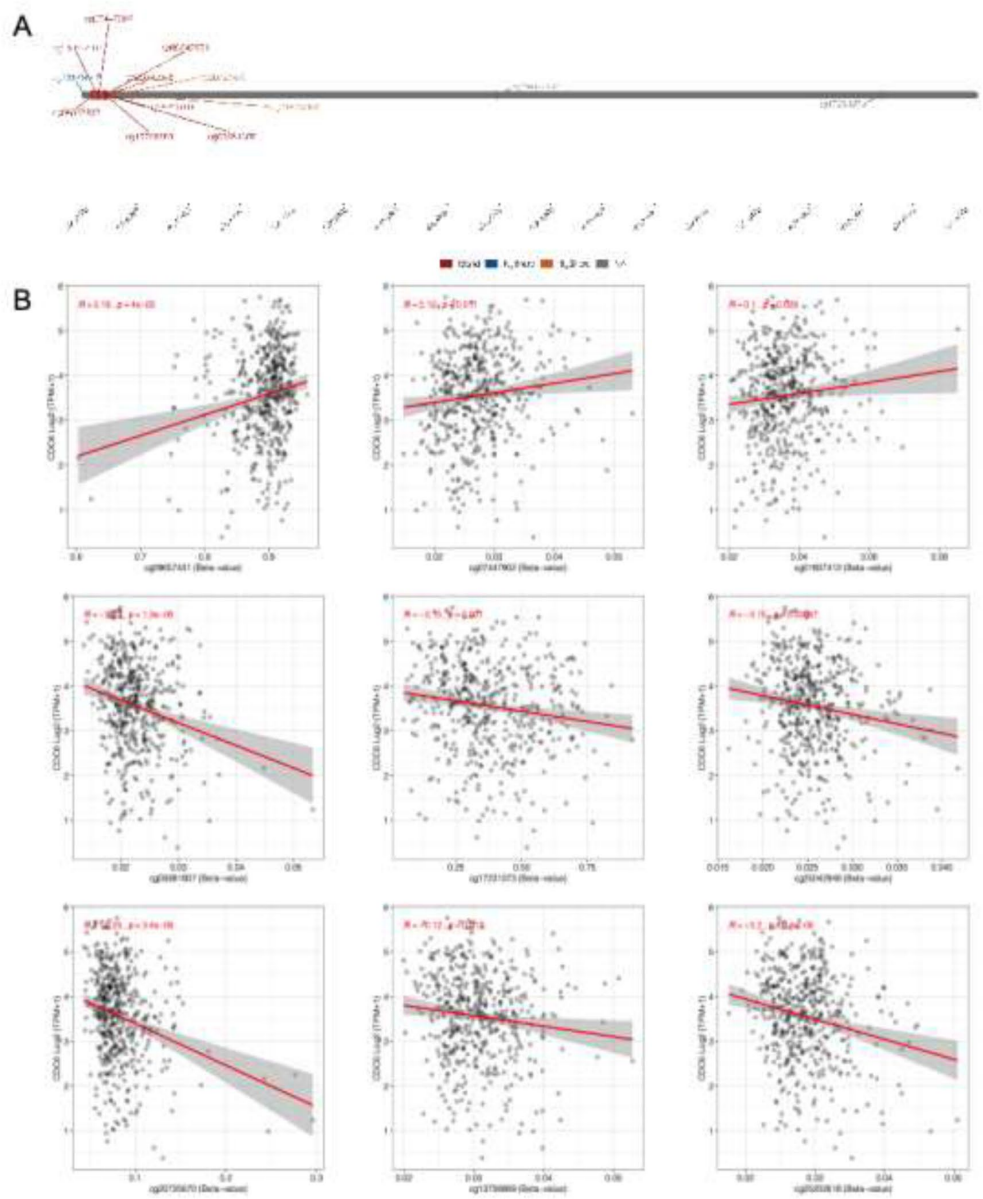
DNA methylation status of CDC6 (**A**) the distribution of 13 methylation sites in CDC6. (**B**) 6 methylation sites (cg05981907, cg17231373, cg25042948, cg20725670, cg13708869, and cg25202618) exhibited a negative correlation with the expression level of CDC6, whereas only 3 methylation sites (cg09657431, cg07447902, and cg01807412) showed a positive correlation with CDC6

DNA methylation, an epigenetic mechanism, can modulate the expression levels of target genes without altering the nucleotide sequence. Figure 6A illustrates the distribution of 13 methylation sites in CDC6. Our analysis revealed that 6 methylation sites (cg05981907, cg17231373, cg25042948, cg20725670, cg13708869, and cg25202618) exhibited a negative correlation with the expression level of CDC6, whereas only 3 methylation sites (cg09657431, cg07447902, and cg01807412) showed a positive correlation with CDC6 (Fig. 6B).

### CDC6 enhances the proliferation, migration, and invasion characteristics of melanoma cells in vitro

**Fig. 7:**
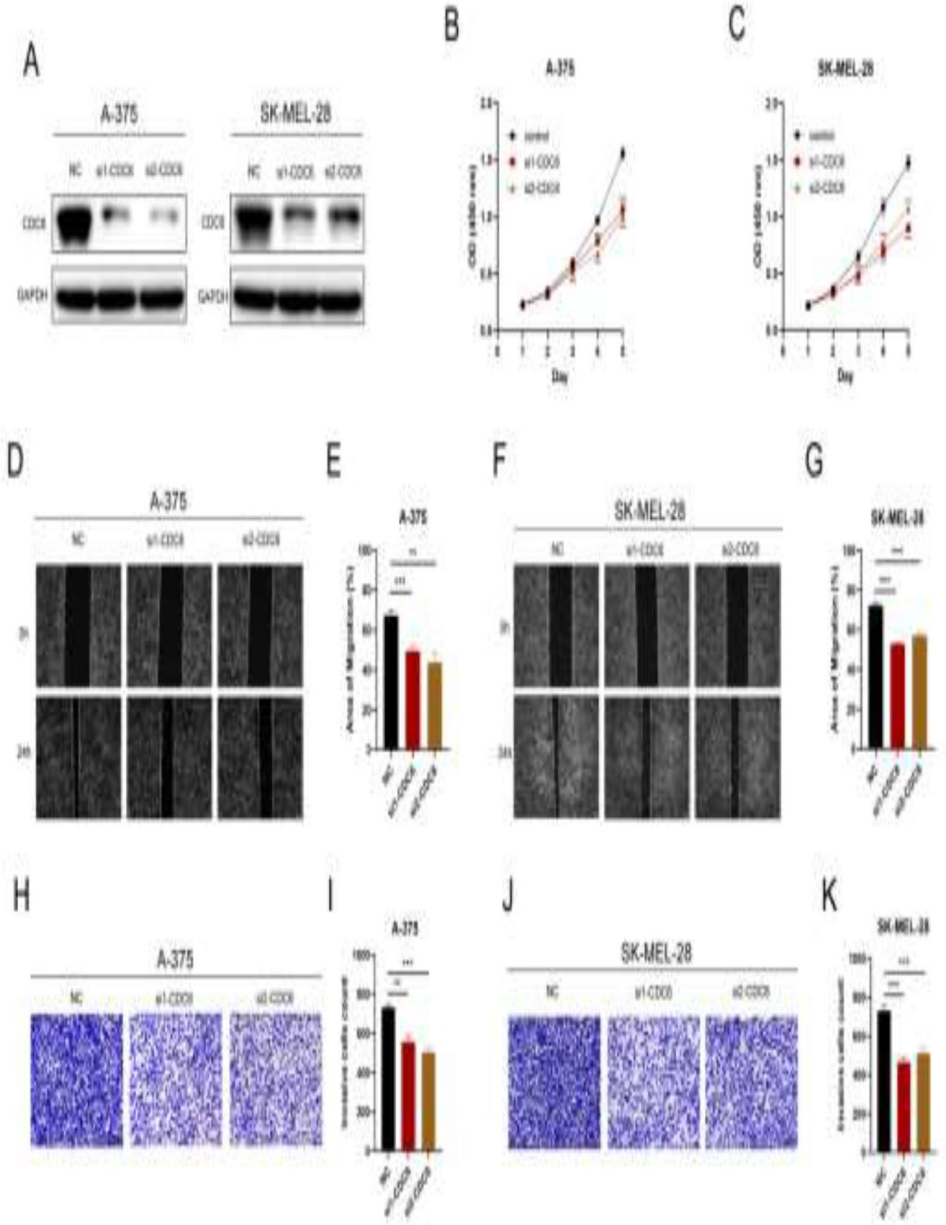
CDC6 enhances the proliferation, migration, and invasion characteristics of melanoma cells in vitro

The biological role of CDC6 in melanoma cells was investigated by in vitro experiments. The siRNAs targeting CDC6 were transfected into human melanoma cells (A-375 and SK-MEL-28), and western blot assay was conducted to verify the transfection efficiency (Fig. 7A). The CCK-8 assay showed that CDC6 knockdown inhibited the proliferative ability of the melanoma cells (Fig. 7B-C). In wound scratch assay, the migrative capacity of the melanoma cells was suppressed by CDC6 knockdown (Fig. 7D-G). Besides, the Transwell assay revealed that the invasive ability of the melanoma cells with CDC6 knockdown was significantly suppressed (Fig. 7H-K). In conclusion, CDC6 accelerates the proliferation, migration, and invasion of melanoma cells in vitro.

### Signaling pathways related to CDC6 in SKCM

**Fig. 8:**
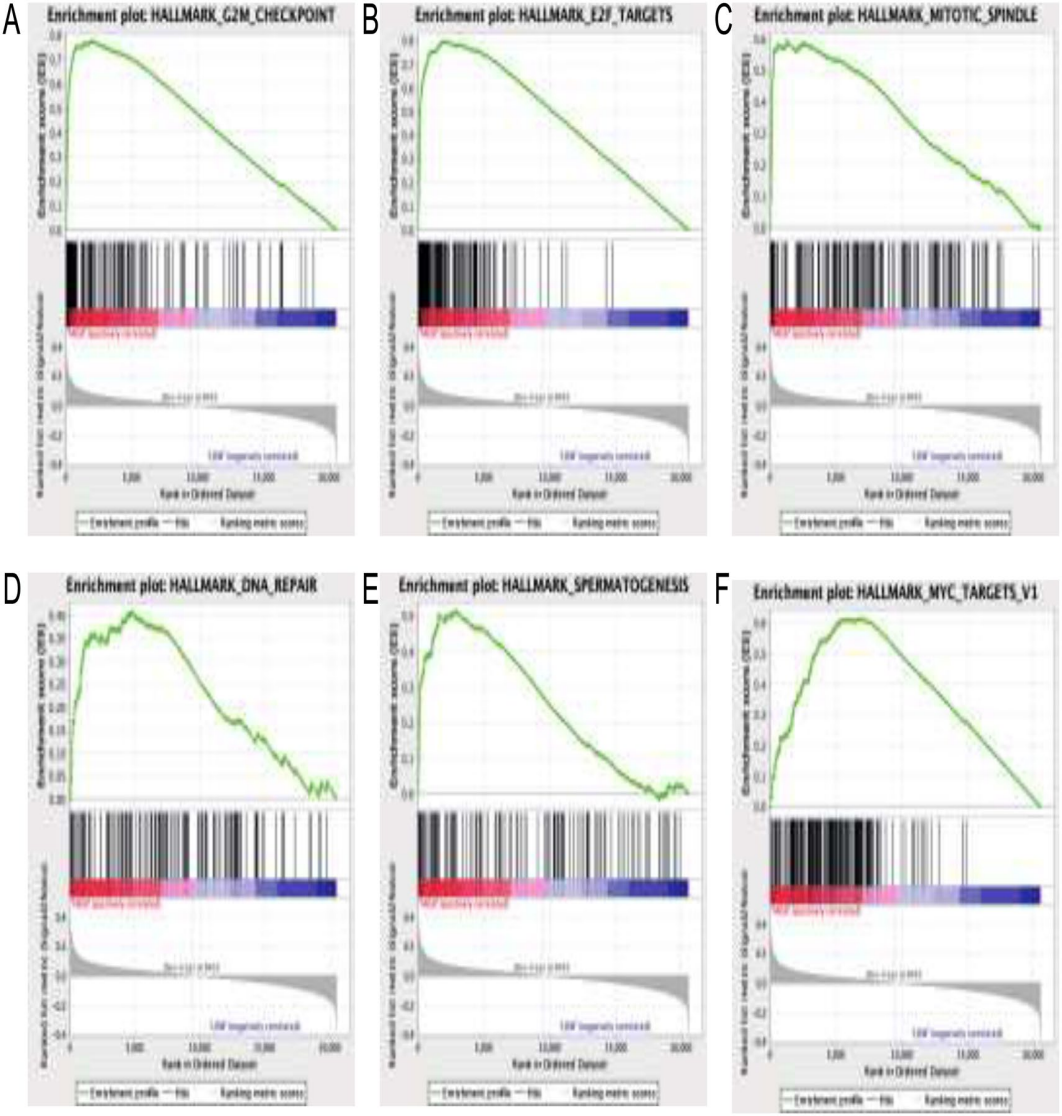
Signaling pathways related to CDC6 in SKCM. GSEA was conducted to pathway analysis, revealing significant enrichment of several cancer-related pathways, including the G2M checkpoint, E2F targets, mitotic spindle, DNA repair, spermatogenesis, and MYC targets pathways

To further elucidate the molecular mechanism underlying CDC6’s impact on prognosis in SKCM, we conducted Gene Set Enrichment Analysis (GSEA) using the TCGA RNA-seq dataset. Our analysis revealed significant enrichment of several cancer-related pathways, including the G2M checkpoint, E2F targets, mitotic spindle, DNA repair, spermatogenesis, and MYC targets pathways, in the high CDC6 expression group (as shown in Fig. 8). These findings suggest that CDC6 may play a role in tumor progression through involvement in these pathways.

### Relationship between CDC6 and immune cell infiltration in SKCM

**Fig. 9:**
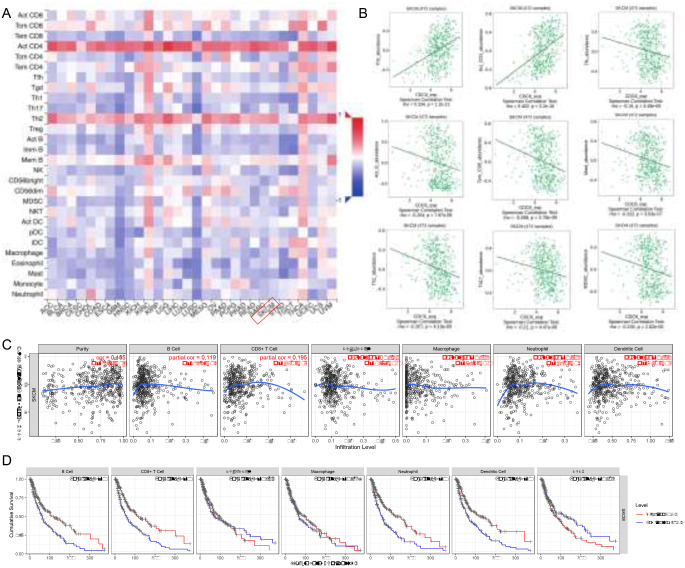
Relationship between CDC6 and immune cell infiltration in SKCM. (**A**) Significant correlations of CDC6 expression with 28 types of tumor-infiltrating lymphocytes (TILs) across various human heterogeneous cancers. (**B**) CDC6 played a crucial role in immune infiltration, exhibiting significant correlations with the abundance of various immune cell types, including Th2 (rho = 0.334, *p* < 0.001), activated CD4 cells (rho = 0.402, *p* < 0.001), Tfh (rho = 0.513, *p* < 0.001), activated B cells (rho = -0.204, *p* < 0.001), Tem_CD8 cells (rho = -0.268, *p* < 0.001), mast cells (rho = -0.232, *p* < 0.001), Th1 (rho = -0.267, *p* < 0.001), Th17 (rho = -0.21, *p* < 0.001), and MDSC (rho = -0.193, *p* < 0.001). (**C**) A positive correlation between the expression of CDC6 and the levels of infiltration of multiple immune cell types (including B cells, CD8 + T cells, CD4 + T cells, macrophages, neutrophils, and dendritic cells) in SKCM. (**D**) Higher infiltration levels of CD4 + T cells, CD8 + T cells, macrophages, neutrophils, B cells, and dendritic cells were associated with a better prognosis in SKCM

To explore the impact of CDC6 expression on the tumor microenvironment in SKCM, we investigated its significant correlations with 28 types of tumor-infiltrating lymphocytes (TILs) across various human heterogeneous cancers (Fig. 9A). As illustrated in Fig. 9B, CDC6 played a crucial role in immune infiltration, exhibiting significant correlations with the abundance of various immune cell types, including Th2 (rho = 0.334, *p* < 0.001), activated CD4 cells (rho = 0.402, *p* < 0.001), Tfh (rho = 0.513, *p* < 0.001), activated B cells (rho = -0.204, *p* < 0.001), Tem_CD8 cells (rho = -0.268, *p* < 0.001), mast cells (rho = -0.232, *p* < 0.001), Th1 (rho = -0.267, *p* < 0.001), Th17 (rho = -0.21, *p* < 0.001), and MDSC (rho = -0.193, *p* < 0.001).

Furthermore, to comprehensively investigate the molecular characteristics of tumor-immune interactions, we utilized the TIMER analysis to assess the relationship between CDC6 and the degree of infiltration of multiple immune cells in SKCM. Our findings revealed a positive correlation between the expression of CDC6 and the levels of infiltration of multiple immune cell types (including B cells, CD8 + T cells, CD4 + T cells, macrophages, neutrophils, and dendritic cells) in SKCM (Fig. 9C). Moreover, we validated that higher infiltration levels of CD4 + T cells, CD8 + T cells, macrophages, neutrophils, B cells, and dendritic cells were associated with a better prognosis in SKCM (Fig. 9D). Therefore, the relationship between SKCM and the tumor immune microenvironment of CDC6 needs further exploration in future research.

## Discussion

SKCM is a significant challenge in oncology due to its aggressiveness. Early detection is crucial for better patient outcomes, as advanced stages have high mortality rates. Currently, melanoma diagnosis relies heavily on histopathological examination, which can vary in interpretation due to the complex nature of melanoma and other skin lesions(Lam et al. 2023). While surgery can effectively treat localized melanomas, metastatic cases present a major challenge in treatment. Managing metastatic melanoma is difficult because of its high mutation rate and the ability of cancer cells to evade the immune system(Villani et al. 2022). Traditional chemotherapy has limited effectiveness. However, recent advances in understanding melanoma and the introduction of immunotherapies, targeted therapies, and combination treatments have offered hope for better outcomes and represent a significant shift in treatment strategies.

Extensive studies have affirmed that DNA methylation plays a crucial role in determining the prognosis of SKCM(Aleotti et al. 2021; Micevic et al. 2017; Wouters et al. 2017). Hence, we conducted an integrated bioinformatics analysis incorporating gene expression and methylation profiling to uncover novel prognostic biomarkers and therapeutic targets in SKCM for future exploration. Our study identified a total of 120 hypomethylated, upregulated genes and 212 hypermethylated, downregulated genes by overlapping DEGs and DMGs. Functional enrichment analysis of the hypomethylation-upregulated genes revealed significant changes in biological processes, particularly enrichment in spindle assembly checkpoint signaling, mitotic spindle assembly checkpoint signaling, mitotic spindle checkpoint signaling, and negative regulation of mitotic metaphase/anaphase transition. GO cell component analysis indicated significant enrichment in heterochromatin, intracellular membrane-bounded organelles, pericentric heterochromatin, and condensed chromosomes. Furthermore, molecular function analysis highlighted significant enrichment in adenyl ribonucleotide binding, DNA secondary structure binding, ATPase activity coupled to the transmembrane movement of ions, and proton-transporting ATPase activity. KEGG pathway enrichment analysis suggested significant enrichment in pathways such as dilated cardiomyopathy, amino sugar and nucleotide sugar metabolism, progesterone-mediated oocyte maturation, and chemical carcinogenesis. A PPI network of hypomethylation-high expression genes illustrated the interactome of hub genes. Subsequently, GEPIA identified the most prognostically relevant hub genes, namely CCNA2, CDC6, CDCA3, CKS2, DTL, HJURP, and NEK2, offering potential insights into therapeutic strategies for SKCM.

After conducting an extensive literature review, we observed that cell division cycle 6 (CDC6) has been relatively underexplored in melanoma research compared to other prognostic genes, despite its implication in the initiation and progression of various human tumors (Wang et al. 2022; Mourkioti et al. 2023; Lim and Townsend 2020). However, its known role in tumor development and prognosis has made CDC6 a focal point of our study. CDC6 plays a key role in controlling DNA replication, making it a focus of research in cancer. In pancreatic cancer, CDC6 interacts with pathways affected by major mutations, like KRAS(Lim and Townsend 2020). It also directly affects processes that promote tumor growth, such as suppressing tumor suppressor genes like CDH1 and influencing cell transition. Gene amplification of CDC6, along with its regulator E2F, is common in tumors with high CDC6 levels, suggesting it plays a significant role in cancer development(Karakaidos et al. 2004). Thus, our study delved into the role of CDC6 in melanoma. We observed elevated expression of CDC6 at both transcriptome and protein levels in melanoma samples, which correlated with poorer outcomes in SKCM patients. Furthermore, through in vitro experiments, we demonstrated that CDC6 enhances the proliferation, migration, and invasion abilities of melanoma cells. We also investigated the impact of methylation status on CDC6 expression using TCGA RNA-seq datasets. Among the identified methylation sites, six (cg05981907, cg17231373, cg25042948, cg20725670, cg13708869, and cg25202618) showed a negative correlation with CDC6 expression, while three (cg09657431, cg07447902, and cg01807412) displayed a positive correlation. The findings suggest that the upregulation of CDC6 in SKCM might result from the decreased methylation status observed at its methylation sites. Notably, GSEA revealed the involvement of several pathways, including G2M checkpoint, E2F targets, mitotic spindle, DNA repair, spermatogenesis, and MYC targets, which are highly relevant to tumor progression, including melanoma. Moreover, CDC6 demonstrated a crucial role in immune infiltration, exhibiting significant correlations with various immune cell abundances, suggesting its potential as an immunotherapeutic target in SKCM.

Regarding the hypermethylation-downregulated genes, functional enrichment analysis revealed significant changes in biological processes, particularly enriched in epidermis development, keratinocyte differentiation, supramolecular fiber organization, and skin development. GO cell component analysis highlighted significant enrichment in cell-cell junctions, intermediate filaments, the cytoskeleton, and intermediate filament cytoskeleton among the downregulated genes. Moreover, for molecular function, the hypermethylation-downregulated genes exhibited significant enrichment in cadherin binding, cadherin binding involved in cell-cell adhesion, chemokine receptor binding, and actin binding. KEGG pathway enrichment analysis indicated significant enrichment in pathways including the estrogen signaling pathway, Staphylococcus aureus infection, axon guidance, and arachidonic acid metabolism. Subsequently, we constructed a PPI network and conducted survival analysis to identify the key prognostic gene among the hypermethylation-downregulated genes. Notably, our investigation highlighted the significance of the keratin(KRT) family in SKCM, with particular emphasis on KRT 5, 14, 15, and 16. This observation is consistent with our prior research findings(Han et al. 2021), reinforcing the pivotal role of these keratins in SKCM pathogenesis.

## Conclusion

To sum up, this study utilized comprehensive bioinformatics analysis to uncover genes and pathways regulated by methylation in SKCM. PPI networks was established and survival analysis was performed to identify the prognostic hub genes. Additionally, we validated the prometastatic role of CDC6 in melanoma and identified its methylation sites, showing the critical role of CDC6 in augmenting the proliferative, migratory, and invasive ability of SKCM. These results contribute to a deeper comprehension of methylation-mediated regulatory mechanisms driving the metastatic potential of primary and metastatic melanoma, offering potential novel biomarkers and therapeutic targets for future investigation.

## Electronic supplementary material

Below is the link to the electronic supplementary material.

Supplementary Figure 1

Supplementary Table 1

## Data Availability

No datasets were generated or analysed during the current study.
