# Supplementary material for: Comprehensive analysis of aberrantly methylated differentially expressed genes and validation of CDC6 in melanoma
Source: J Cancer Res Clin Oncol. 2024 Jul 25;150(7):362. doi: 10.1007/s00432-024-05851-x (PMC11272740; doi:10.1007/s00432-024-05851-x)

**Supplementary Fig. 1.** IHC staining indicated significantly elevated CDC6 expression in terms of density and intensity in melanoma tissues compared with normal skin tissues. IHC score between melanoma and normal skin tissues were illustrated (P < 0.0001).


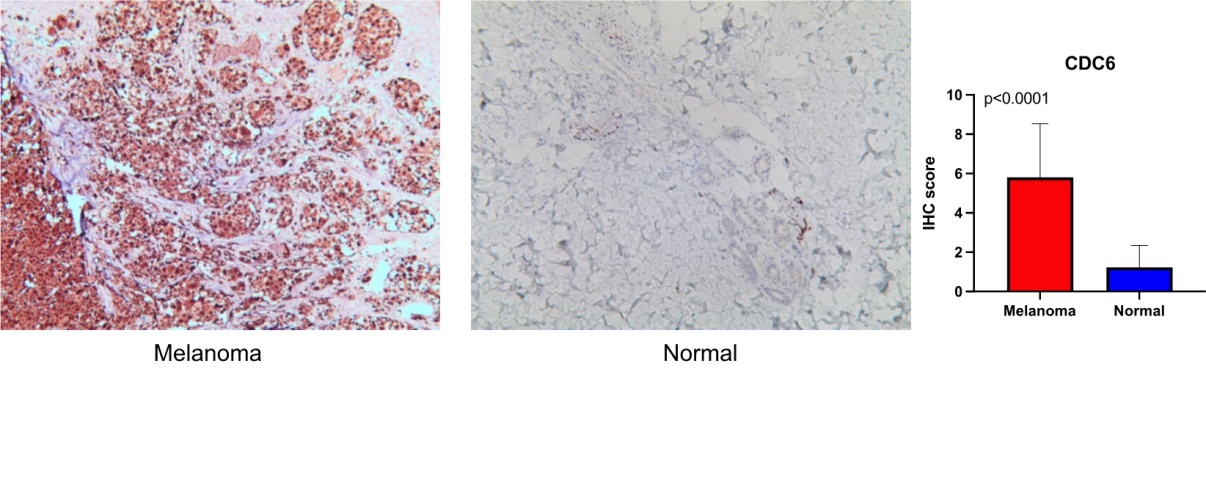

Supplement: Supplementary file 1 — Supplementary Figure 1 [file 432_2024_5851_MOESM1_ESM.docx]
