# Supplementary material for: Comprehensive analysis of aberrantly methylated differentially expressed genes and validation of CDC6 in melanoma
Source: J Cancer Res Clin Oncol. 2024 Jul 25;150(7):362. doi: 10.1007/s00432-024-05851-x (PMC11272740; doi:10.1007/s00432-024-05851-x)
Supplement: Supplementary file 2 — Supplementary Table 1 [file 432_2024_5851_MOESM2_ESM.docx]

| Table S1. The sequences of siRNAs | | |  |
| --- | --- | --- | --- |
|  |  |  |  |
| Target gene | Type | No. | Sequence |
| CDC6 | siRNA | 1 | 5′-AACUUCCCACCUUAUACCAGA-3′ |
|  |  | 2 | 5′-AAGAAUCUGCAUGUGUGAGAC-3′ |
